# The emerging role of brain-derived microparticles in trauma-induced coagulopathy after traumatic brain injury

**DOI:** 10.3389/fneur.2026.1904648

**Published:** 2026-09-25

**Authors:** Xinrong Cai, Han She, Qingxiang Mao

**Affiliations:** Department of Anesthesiology, Daping Hospital, Army Medical University, Chongqing, China

**Keywords:** biomarker, blood-brain barrier, brain-derived microparticles, coagulopathy, extracellular vesicles, neuroinflammation, therapeutic target, trauma-induced coagulopathy

## Abstract

Traumatic brain injury (TBI) is frequently complicated by trauma-induced coagulopathy (TIC), which contributes substantially to secondary brain injury and poor clinical outcomes. The mechanisms underlying traumatic brain injury induced coagulopathy (TBI-IC) are multifactorial and involve extensive cellular alterations in endothelial cells, neutrophils, and platelets. Brain-derived microparticles (BDMPs), a subgroup of extracellular vesicles derived from brain-associated cells, attracted increasing attention due to their potential involvement in coagulation regulation after TBI. This review summarizes current evidence regarding the biological characteristics of BDMPs, their proposed contributions to coagulation abnormalities, inflammatory responses, and vascular dysfunction following TBI, as well as their potential value as biomarkers and therapeutic targets. In addition, the limitations of current evidence, including methodological heterogeneity, insufficient clinical validation, and uncertainties regarding causal mechanisms, are discussed to provide a balanced perspective on the translational potential of BDMPs in TBI-IC.

## 1 Introduction

Traumatic brain injury (TBI) is a major cause of mortality and long-term disability worldwide. Approximately half of patients with TBI may develop coagulation abnormalities, which are closely associated with secondary neurological deterioration and adverse outcomes (1). Following the initial mechanical insult, TBI triggers a series of secondary pathological responses involving systemic inflammation, neuronal injury, glial activation, vascular dysfunction, and disturbances in hemostasis (2, 3).

TBI-induced coagulopathy (TBI-IC) is a dynamic and heterogeneous process rather than a uniform transition between hypercoagulation and hypocoagulation (4). Early after injury, coagulation factor consumption, endothelial activation, and excessive fibrinolysis may contribute to bleeding tendency, whereas hypercoagulability and fibrinolytic shutdown may occur during subsequent phases (5–7). The temporal pattern and dominant phenotype may vary depending on injury severity, associated systemic trauma, experimental conditions, and clinical management strategies (8).

Although conventional coagulation abnormalities after TBI have traditionally been attributed to coagulation factor consumption, platelet dysfunction, endothelial injury, and inflammatory activation, increasing evidence suggests that extracellular vesicle-mediated intercellular communication may also participate in this process (9, 10).

Brain-derived microparticles (BDMPs) are a subgroup of extracellular vesicles generated from brain-associated cells under physiological or pathological conditions (11). Unlike soluble mediators, BDMPs contain membrane-associated proteins, lipids, and nucleic acids derived from their parent cells, enabling them to influence recipient cells through direct interaction or molecular cargo transfer (11). Following TBI, neuronal injury, glial activation, and disruption of the neurovascular unit may promote BDMP release and circulation (12). Increasing studies have reported altered circulating BDMP levels after TBI and their association with injury severity and coagulation abnormalities (13). However, current evidence remains largely derived from experimental studies, and whether BDMPs directly contribute to TBI-IC or primarily reflect tissue injury remains incompletely understood (14).

This review summarizes current knowledge regarding BDMP biology, their proposed involvement in TBI-IC, their potential clinical application as biomarkers, and emerging therapeutic strategies targeting BDMP-related pathways. Particular emphasis is placed on evidence limitations, unresolved controversies, and challenges associated with clinical translation.

### 1.1 Literature search strategy

A literature search was conducted using PubMed, Web of Science, and Embase databases to identify relevant studies investigating the biological characteristics, pathological mechanisms, biomarker potential, and therapeutic implications of BDMPs in TBI-IC. The search terms included combinations of “brain-derived microparticles,” “brain-derived extracellular vesicles,” “microparticles,” “extracellular vesicles,” “traumatic brain injury,” “trauma-induced coagulopathy,” “coagulopathy,” “coagulation,” “inflammation,” “blood-brain barrier,” “biomarker,” and “therapy.” Studies published from database inception to June 2025 were considered. Original research articles, clinical studies, experimental studies, systematic reviews, and relevant guidelines were included when they provided information related to BDMP biology, EV-mediated mechanisms, coagulation abnormalities, clinical applications, or therapeutic strategies after TBI. Studies unrelated to TBI, BDMPs, extracellular vesicle biology, or coagulation mechanisms were excluded. Because BDMP-specific clinical evidence remains limited, studies investigating broader extracellular vesicle populations were considered when they provided mechanistic insights relevant to BDMP biology, with their limitations explicitly discussed throughout the review.

## 2 Biogenesis and molecular characteristics of BDMPs

### 2.1 Origins and biogenesis of BDMPs

BDMPs represent a specific subgroup of extracellular vesicles (EVs) originating from brain-associated cells (11). According to current extracellular vesicle classification criteria, BDMPs mainly correspond to plasma membrane-derived microparticles or microvesicles and should be distinguished from small extracellular vesicles (sEVs, historically referred to as exosomes) and apoptotic bodies based on their biogenesis pathways and biological characteristics (11, 15). Small extracellular vesicles are generally generated through intracellular endosomal pathways, whereas microparticles are primarily formed through outward budding and shedding of the plasma membrane (16). In contrast, apoptotic bodies are larger vesicular structures released during late-stage apoptosis and cellular fragmentation (17).

BDMPs were initially identified in brain extracellular environments and were subsequently recognized as potential mediators of intercellular communication within the central nervous system. Multiple brain-associated cell types, including neurons, astrocytes, oligodendrocytes, microglia, and cerebrovascular endothelial cells, have been reported to contribute to brain-derived extracellular vesicle populations (18, 19). However, the relative contribution of each cellular source to circulating BDMPs after TBI remains incompletely defined.

Under physiological conditions, extracellular vesicle release contributes to cellular communication and tissue homeostasis. In pathological states, including oxidative stress, inflammation, hypoxia, and cellular injury, vesicle production and release may be enhanced (15, 17, 20). Microparticles are typically generated through plasma membrane remodeling, cytoskeletal rearrangement, and membrane blebbing processes (16, 21). These events are regulated by multiple molecular pathways, including calcium-dependent signaling, cytoskeletal regulators, membrane lipid redistribution, and vesicle-associated release machinery (22, 23).

Following TBI, several pathological processes may promote BDMP generation. Mechanical injury directly disrupts neuronal and glial cell membranes, resulting in intracellular calcium influx and activation of calcium-dependent enzymes such as calpain (14, 22, 24). These changes may promote cytoskeletal remodeling and membrane shedding, facilitating microparticle formation (22). In addition, damage-associated molecular patterns (DAMPs), inflammatory signaling, and oxidative stress may further enhance extracellular vesicle release by activating innate immune pathways and cellular stress responses (25, 26).

Neuroinflammation represents another potential contributor to BDMP production after TBI. Activated microglia, astrocytes, and infiltrating immune cells release inflammatory mediators, including tumor necrosis factor-α TNF−α and interleukin-1β IL−1β , which may regulate extracellular vesicle biogenesis through inflammatory signaling pathways (27). Reactive oxygen species (ROS) and other cellular stress responses induced by TBI may also influence vesicle secretion as part of cellular stress adaptation (28).

Disruption of the neurovascular unit and blood-brain barrier (BBB) integrity may facilitate the transition of brain-derived vesicles from the injured brain tissue into the systemic circulation (13, 29). In addition, injured cerebrovascular endothelial cells themselves may release endothelial-derived microparticles, which may coexist with and contribute to circulating extracellular vesicle populations after TBI (29). However, distinguishing BDMPs derived from specific brain cell types from other circulating microparticle populations remains technically challenging.

Therefore, although current studies suggest that TBI enhances BDMP release and systemic dissemination, the precise mechanisms regulating BDMP generation, cellular origin, and temporal dynamics remain incompletely understood. Experimental studies have demonstrated that BDMPs can induce systemic coagulation after TBI, while clinical studies have detected altered circulating BDMP levels in patients with severe TBI (13, 14). Further studies using standardized isolation and characterization approaches are required to clarify the biological significance of BDMPs in TBI-associated pathological responses.

### 2.2 Molecular composition and functional characteristics of BDMPs

Thebiological functions of BDMPs are largely determined by their membrane composition and transported molecular cargo. Similar to other extracellular vesicles, BDMPs contain diverse lipids, proteins, and nucleic acids derived from their parental cells, allowing them to participate in intercellular communication. The major molecular components, cellular origins, and potential biological functions of BDMPs are summarized in Table 1 (11). However, the molecular composition of BDMPs is highly heterogeneous and may vary according to cellular origin, injury severity, and disease stage (17).

**Table 1:** Molecular characteristics evidence sources, and proposed functions of BDMP subpopulations.

Subpopulation	Cellular origin	Representative markers	Proposed functions	Evidence source
Neuron-derived BDMPs	Neurons	Tau, NSE, miR- 124	Potential indicators of neuronal injury; possible regulation of coagulation/inflammation	Experimental studies
Astrocyte-derived BDMPs	Astrocytes	GFAP, AQP4, miR-21	Associated with BBB dysfunction and inflammatory responses	Experimental/clinical association studies
Microglia-derived BDMPs	In review Microglia	IL- 1β, HMGB1	Associated with BBB dysfunction and inflammatory responses	Experimental studies
Mitochondrial microparticles (mtMPs)	Neurons/glia	Cardiolipin, mtDNA	Possible involvement in coagulation and inflammation	Mainly experimental studies
Endothelial cell-derived BDMPs	Cerebrovascular endothelial cells	P-selectin, vWF	Possible contribution to vascular activation	Experimental studies

#### 2.2.1 Membrane lipid components

The membrane structure of BDMPs contains various phospholipids, cholesterol, sphingolipids, and lipid raft-associated molecules (10, 30). Among these components, phosphatidylserine (PS) exposure on the outer leaflet of microparticle membranes has attracted considerable attention because of its potential role in coagulation regulation (11, 31).

Under normal physiological conditions, PS is mainly restricted to the inner leaflet of the plasma membrane. During cellular activation, apoptosis, or microparticle formation, membrane asymmetry may be disrupted, resulting in externalization of PS. The negatively charged PS surface can provide a platform for assembly of coagulation-related complexes and may enhance thrombin generation (32, 33).

Because brain-derived cells contain abundant membrane phospholipids, BDMPs may carry relatively high levels of PS compared with some other extracellular vesicle populations. Experimental studies have suggested that PS exposure contributes to the procoagulant activity of microparticles (34, 35). In the context of TBI, brain-derived microparticles have been shown to induce systemic coagulation responses, suggesting that BDMP-associated PS exposure may contribute to their procoagulant properties (14). However, the relative contribution of BDMP-associated PS compared with other circulating PS-positive microparticles in human TBI remains unclear.

In addition to PS, lipid raft-associated components such as cholesterol, sphingomyelin, and ceramide may participate in membrane organization and signaling regulation. These lipid components may influence vesicle stability, cargo selection, and interactions with recipient cells (17). Acid sphingomyelinase-dependent ceramide generation has also been implicated in microparticle release from glial cells, suggesting a potential role of sphingolipid metabolism in brain-derived microparticle formation (26).

#### 2.2.2 Protein components

BDMPs contain membrane-associated proteins and intravesicular proteins that reflect their cellular origin and biological functions. Among membrane proteins, tissue factor (TF) is considered one of the most important molecules associated with microparticle-mediated coagulation regulation. TF-positive microparticles may promote activation of the extrinsic coagulation pathway by facilitating factor VII/VIIa-dependent signaling and subsequent thrombin generation (34, 36, 37).

However, whether BDMP-derived TF represents a dominant source of circulating TF activity after TBI remains uncertain. Current evidence supporting TF-mediated BDMP effects mainly comes from experimental models, whereas the contribution of different cellular sources of TF in patients with TBI requires further clarification.

BDMPs may also carry brain-derived proteins, which may reflect their cellular origin and potentially serve as indicators of brain injury. In patients with severe isolated TBI, circulating BDMPs expressing glial fibrillary acidic protein (GFAP), neuron-specific enolase (NSE), and aquaporin-4 (AQP4) have been detected, supporting their potential use as indicators of brain cellular injury (13).

In addition, brain-derived extracellular vesicle populations have been reported to contain neuronal and axonal injury-associated proteins, including Tau, neurofilament light chain (NfL), and ubiquitin carboxy-terminal hydrolase L1 (UCH-L1), although whether these molecules are specifically enriched in BDMP populations remains unclear (38–40).

Furthermore, proteomic analyses of extracellular vesicle and microparticle populations have identified inflammatory and coagulation-associated proteins, including fibrinogen-related proteins, PAI-1, complement components, and HMGB1 (41–43). These molecules may participate in inflammatory and coagulation regulation; however, their specific enrichment and functional contribution within BDMP populations requires further validation.

#### 2.2.3 Nucleic acid cargo

BDMPs can transport various nucleic acid molecules, including microRNAs (miRNAs), messenger RNAs (mRNAs), and long non-coding RNAs (lncRNAs) (44). Through delivery of these molecules, extracellular vesicles may influence gene expression and cellular responses in distant tissues (11, 20).

Following TBI, several miRNAs enriched in brain-derived vesicles have been implicated in regulation of inflammation, BBB integrity, oxidative stress, and cellular injury responses. For example, miR-124 and miR-21 have been investigated as potential regulators of neuronal injury and inflammatory signaling (45).

However, current studies have mainly examined extracellular vesicle-associated miRNA profiles rather than specifically isolated BDMP populations. Therefore, whether these molecular signatures are unique characteristics of BDMPs or represent broader EV responses remains uncertain.

#### 2.2.4 Mitochondrial-derived microparticles

Mitochondrial components represent another important cargo type identified in extracellular vesicle populations after brain injury. Mitochondrial microparticles (mtMPs) may contain mitochondrial DNA, mitochondrial proteins, or other mitochondrial components released from injured neurons and glial cells (46–48).

Experimental studies have suggested that mitochondrial components, including cardiolipin, may participate in coagulation regulation and inflammatory activation after TBI. Cardiolipin exposure on mitochondrial-derived vesicles has been reported to influence coagulation-related pathways and platelet activation (46, 49, 50).

Nevertheless, the contribution of mtMP-associated mitochondrial components to human TBI-IC remains incompletely understood. Most current evidence is derived from animal models, and further clinical studies are required to determine whether mtMPs represent independent pathogenic mediators or biomarkers of mitochondrial injury.

#### 2.2.5 Dynamic changes in BDMP composition after TBI

Importantly, the molecular composition of BDMPs is not static. Following TBI, different stages of injury may be associated with distinct vesicle profiles. Early after injury, BDMPs may contain more damage-associated molecules and procoagulant components, whereas later changes may involve immune regulation and tissue repair-related signals.

Experimental studies have demonstrated that TBI alters the quantity and biological activity of brain-derived extracellular vesicles, which may contribute to systemic inflammatory and coagulation responses (14, 51).

The heterogeneity of BDMP composition provides a potential explanation for their diverse biological effects but also represents a major challenge for clinical translation. Comprehensive characterization of BDMP subpopulations based on cellular origin, molecular cargo, and functional activity will be essential for understanding their role in TBI-IC.

## 3 Pathophysiological role of BDMPs in TBI-IC

BDMPs have been proposed to participate in TBI-IC through multiple interconnected mechanisms, including coagulation regulation, platelet modulation, inflammatory amplification, disruption of the neurovascular unit, and alteration of fibrinolytic balance (52) (Figure 1). Current evidence supporting these mechanisms is derived predominantly from experimental models, whereas available clinical studies have mainly demonstrated associations between circulating extracellular vesicle levels and injury severity (13, 14, 51). Therefore, although BDMPs represent a potential mediator linking brain injury and systemic coagulation abnormalities, their direct causal contribution to TBI-IC remains to be fully established.

**Figure 1:**
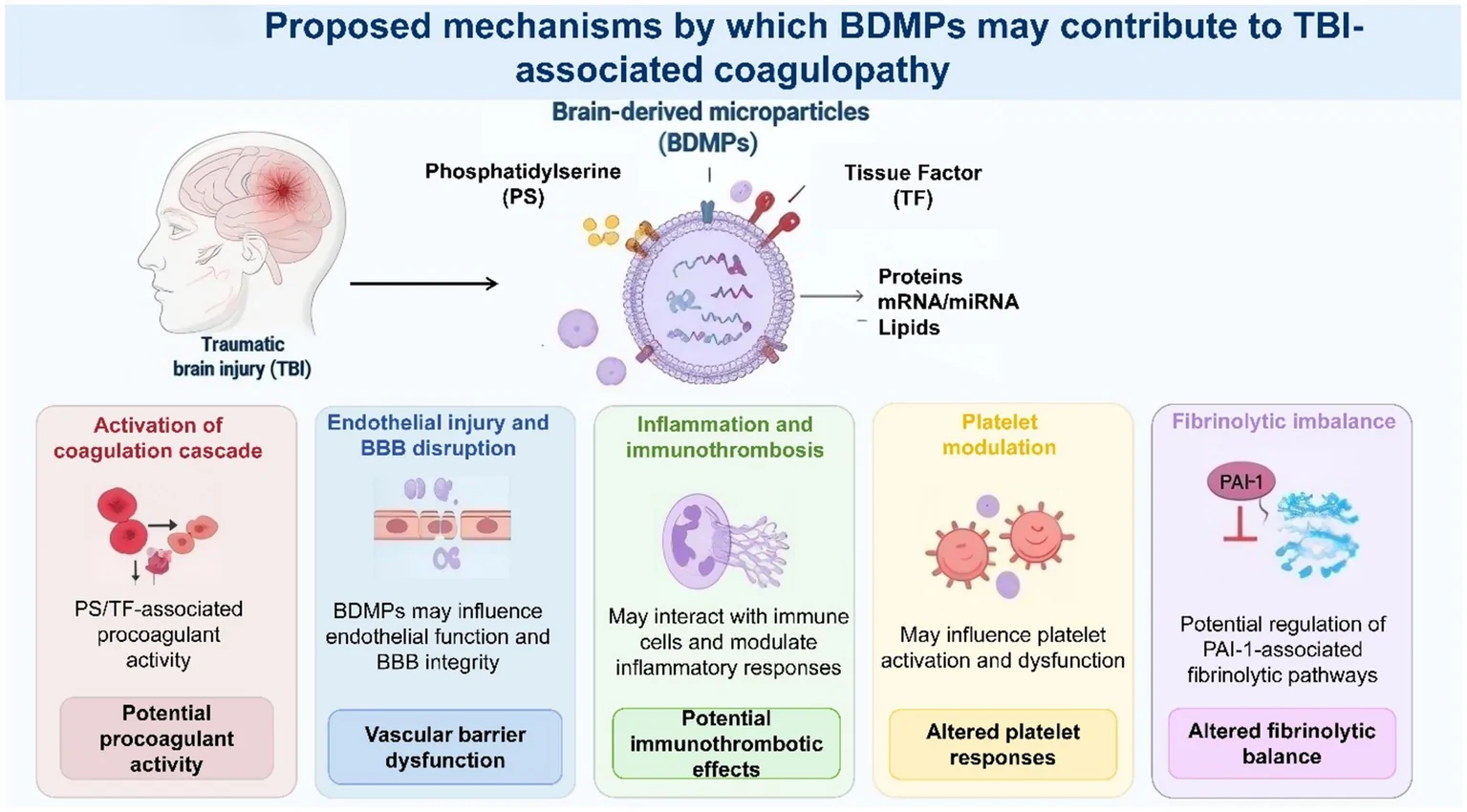
Proposed mechanisms by which BDMPs may contribute to TBI-IC. Following traumatic brain injury, BDMP release from injured brain-associated cells may influence systemic coagulation through multiple potential pathways, including coagulation regulation, platelet modulation, inflammatory responses, vascular dysfunction, and altered fibrinolytic balance. Current evidence supporting these mechanisms is mainly derived from experimental studies, and their clinical relevance requires further validation.

The biological effects of BDMPs are likely influenced by their cellular origin, molecular cargo, temporal release pattern, and interaction with circulating blood components (53). Rather than acting through a single pathway, BDMPs may represent a heterogeneous population of injury-associated vesicles that participate in multiple pathological processes after TBI (11, 17).

### 3.1 BDMPs and activation of the coagulation system

One of the most extensively investigated functions of microparticles is their potential procoagulant activity. BDMPs contain exposed PS and may express TF, providing a negatively charged surface that facilitates assembly of coagulation complexes and promotes thrombin generation (32, 34). Experimental studies have suggested that BDMP-associated PS and TF may contribute to coagulation activation following TBI (14, 51, 54).

However, the presence of procoagulant components on BDMPs does not necessarily indicate that BDMPs are the primary initiators of coagulation abnormalities in patients with TBI. Multiple sources of circulating microparticles, including activated platelets, endothelial cells, and immune cells, may also contribute to systemic coagulation activation (25, 34, 55). Therefore, the relative contribution of BDMPs compared with other circulating microparticle populations remains uncertain.

Following TBI, BDMP levels have been reported to increase rapidly and reach peak concentrations within several hours after injury (13, 14). In contrast, experimental and clinical observations suggest that some coagulation abnormalities may become more prominent at later stages, including approximately 24 h after injury (5, 6). This temporal discrepancy indicates that BDMPs may function as early signaling mediators or amplifiers of coagulation responses rather than directly reflecting the coagulation phenotype at a single time point.

BDMP-associated TF may activate the extrinsic coagulation pathway through interaction with factor VII/VIIa and subsequently promote thrombin formation (34, 36, 56). Nevertheless, the specific contribution of BDMP-derived TF compared with TF released from other cellular sources remains unclear, particularly in human TBI. Further studies using cell-specific markers and functional assays are required to determine whether BDMP-associated TF represents a major determinant of trauma-induced coagulation abnormalities.

In addition to the extrinsic coagulation pathway, some studies have suggested that extracellular vesicle-associated components may activate the contact pathway through factor XII. Although factor XII activation represents a possible mechanism linking extracellular vesicles with coagulation initiation, its clinical relevance in TBI-IC remains controversial (57). Whether factor XII-dependent signaling represents a primary pathogenic pathway or an amplification mechanism requires further investigation.

Overall, current evidence supports the concept that BDMPs possess procoagulant potential; however, the extent to which they directly contribute to clinical coagulopathy after TBI remains unresolved (14, 58).

### 3.2 BDMPs and platelet regulation

Platelet abnormalities are common after TBI and represent an important component of trauma-induced coagulopathy. BDMPs may interact with platelets through surface molecules, lipid components, and associated signaling pathways, thereby influencing platelet activation and functional responses (59–61).

Experimental studies have demonstrated that microparticles can promote platelet activation by facilitating platelet adhesion, aggregation, and surface receptor signaling (22, 61, 62). BDMPs carrying PS and other bioactive molecules may therefore contribute to platelet activation under pathological conditions (14, 34).

However, platelet responses after TBI are complex and cannot be simply characterized as excessive activation. Clinical observations indicate that platelet activation, impaired platelet aggregation, and thrombocytopenia may occur simultaneously after injury (59, 63). This apparent paradox suggests that platelet activation and platelet dysfunction represent different aspects of a dynamic pathological process.

Persistent activation of platelets may induce structural and functional changes, including increased PS exposure, enhanced clearance, and exhaustion of platelet responses. These changes may contribute to reduced platelet numbers and impaired hemostatic function during later stages of injury (62, 64).

Therefore, BDMP-mediated platelet regulation may be highly dependent on injury stage, inflammatory environment, and interaction with other circulating factors. Further investigation is required to clarify whether BDMPs primarily enhance platelet activation or contribute to platelet dysfunction during different phases of TBI-IC.

### 3.3 BDMPs and inflammatory amplification

Beyond coagulation regulation, BDMPs may participate in inflammatory responses following TBI. Extracellular vesicles can transport inflammatory mediators, nucleic acids, and damage-associated molecular patterns (DAMPs), allowing communication between injured brain tissue and peripheral immune cells (11, 25).

Following TBI, BDMPs may interact with immune cells, including monocytes, macrophages, and endothelial cells, and potentially influence inflammatory signaling pathways (12, 51, 65). Vesicle-associated molecules such as HMGB1 and inflammatory mediators may contribute to amplification of systemic inflammatory responses.

Nevertheless, distinguishing BDMP-specific inflammatory effects from broader extracellular vesicle-mediated responses remains challenging. Many studies investigating inflammatory functions have examined total extracellular vesicle populations rather than specifically isolated BDMPs (11, 25). Therefore, whether BDMPs possess unique inflammatory properties compared with other extracellular vesicles remains unclear.

### 3.4 BDMP and blood-brain barrier disruption

The blood-brain barrier (BBB) represents an important interface between the injured brain and systemic circulation. TBI-induced BBB disruption facilitates the release of brain-derived components into peripheral blood and may also allow circulating inflammatory mediators to influence cerebral injury (66–68).

BDMPs have been implicated in BBB dysfunction through several potential mechanisms, including regulation of endothelial signaling, modulation of tight junction proteins, and interaction with matrix metalloproteinases (MMPs) (29, 69, 70). Experimental studies suggest that BDMP-associated signals may influence MMP-9 activity and contribute to alteration of endothelial integrity (69).

However, current evidence supporting BDMP-mediated BBB disruption is mainly derived from experimental models. The extent to which circulating BDMPs contribute to BBB dysfunction in human TBI remains unclear.

Furthermore, BBB disruption itself may promote additional BDMP release by increasing communication between damaged brain tissue and the circulation, suggesting a potential bidirectional relationship between BBB injury and BDMP generation (40, 68).

### 3.5 BDMPs and fibrinolytic dysregulation

Abnormal fibrinolysis represents another major feature of TBI-IC. Early after severe trauma, excessive fibrinolysis may contribute to bleeding tendency, whereas later suppression of fibrinolysis, known as fibrinolytic shutdown, may promote thrombotic complications (2, 6).

BDMPs may participate in this dynamic process through transport of antifibrinolytic molecules, including plasminogen activator inhibitor-1 (PAI-1) (71). By influencing endothelial fibrinolytic signaling and coagulation balance, BDMPs may contribute to reduced fibrinolytic activity after TBI (25, 36).

However, fibrinolytic regulation after TBI is controlled by multiple interacting mechanisms, including endothelial activation, inflammatory responses, tissue hypoperfusion, and systemic coagulation changes (72, 73). The relative contribution of BDMP-associated PAI-1 compared with other systemic sources remains uncertain.

Therefore, BDMPs may represent one component of the complex transition from early hyperfibrinolysis to subsequent fibrinolytic shutdown, but further studies are needed to establish direct causal relationships (2, 8).

## 4 Biomarker potential of BDMPs

### 4.1 BDMPs as candidate biomarkers in TBI

The identification of reliable biomarkers capable of reflecting brain injury severity, coagulation abnormalities, and clinical prognosis remains an important challenge in the management of TBI (74–76). Due to their origin from injured brain-associated cells and their ability to enter peripheral circulation following disruption of the neurovascular unit, BDMPs have attracted increasing interest as potential biomarkers for TBI assessment (13, 18, 40).

Compared with conventional soluble biomarkers, extracellular vesicles possess several theoretical advantages, including protection of molecular cargo from degradation and the ability to reflect cellular responses (11, 77). Therefore, circulating BDMPs may provide additional information regarding cellular injury, inflammatory activation, and vascular dysfunction after TBI (25, 51).

However, current evidence supporting BDMPs as clinical biomarkers remains preliminary. Most available studies have evaluated extracellular vesicle populations or microparticle levels in general rather than specifically characterized BDMP subpopulations (11, 78). Although increased circulating extracellular vesicle levels have been associated with injury severity and adverse outcomes after TBI, whether BDMP-specific alterations provide additional diagnostic or prognostic value remains uncertain (13, 74).

Furthermore, elevated BDMP levels may represent a response to brain tissue injury rather than an independent driver of disease progression. Therefore, BDMPs should currently be considered candidate biomarkers requiring further validation rather than established clinical indicators (13).

### 4.2 Association between BDMP levels and TBI severity

Several studies have reported increased circulating extracellular vesicle concentrations following TBI, with higher levels observed in patients with more severe neurological injury. These findings suggest that extracellular vesicle abundance may reflect the extent of tissue damage and systemic response after trauma (12, 13).

However, interpretation of these findings requires caution. Most clinical studies have measured total extracellular vesicle populations without sufficient discrimination between different cellular origins (11, 78). Because circulating vesicles may originate from platelets, endothelial cells, immune cells, and damaged brain tissue, the specific contribution of BDMPs to these observations remains unclear (25, 53).

The clinical relevance of BDMP measurement may depend not only on total vesicle abundance but also on vesicle phenotype, including cellular markers, molecular cargo, and functional activity (11, 18). Future studies should focus on identifying BDMP-specific signatures that distinguish brain-derived vesicles from other circulating extracellular vesicle populations (40).

In addition, prospective longitudinal studies are needed to determine whether temporal changes in BDMP levels correlate with disease progression, coagulation abnormalities, neurological deterioration, or treatment response (5, 13).

Although BDMP-related biomarkers show potential for reflecting TBI severity and coagulation-associated changes, current evidence remains insufficient for routine clinical application. Representative studies investigating BDMP-associated biomarkers, detection methods, and biological implications are summarized in Table 2.

**Table 2:** Current evidence and limitations of proposed BDMP-related mechanisms in TBI-associated coagulopathy.

Proposed mechanism	Current evidence	Evidence limitation
Coagulation activation	Mainly animal and experimental studies demonstrating PS/TF-associated procoagulant activity	Limited human causal evidence; contribution compared with other microparticles unclear
Platelet regulation	Experimental studies showing interaction between microparticles and platelet activation pathways	Activation and dysfunction may coexist; disease-stage effects remain unclear
Inflammatory amplification	Experimental studies involving EV-mediated immune regulation	BDMP-specific effects difficult to distinguish from general EV effects
BBB disruption	Mainly experimental evidence involving endothelial injury and MMP pathways	Lack of direct clinical validation
Fibrinolytic regulation	Experimental and association studies involving PAI-1 and fibrinolytic pathways	Mechanistic contribution remains uncertain

### 4.3 Potential application of BDMPs in diagnosis and prognostic prediction

BDMPs may theoretically provide complementary information for several clinical purposes, including early injury assessment, prediction of secondary complications, and evaluation of disease progression.

First, because BDMP release may occur shortly after cellular injury, early changes in circulating BDMP levels could potentially reflect ongoing damage to the neurovascular unit before conventional clinical manifestations become apparent (13, 14).

Second, BDMP-associated molecular signatures may provide information regarding specific pathological processes, such as endothelial dysfunction, inflammatory activation, and coagulation imbalance (29, 51). Therefore, BDMP characterization may offer a biological perspective beyond routine coagulation tests.

Third, changes in BDMP profiles during hospitalization may potentially reflect dynamic alterations in disease state and therapeutic response.

Nevertheless, these applications remain hypothetical. Current clinical evidence is insufficient to determine whether BDMP measurement improves patient stratification beyond established parameters, including Glasgow Coma Scale (GCS), imaging findings, conventional coagulation tests, fibrinogen levels, platelet counts, and viscoelastic hemostatic assays (79–81).

### 4.4 Technical challenges and clinical feasibility of BDMP detection

Despite their potential clinical value, several methodological challenges limit the translation of BDMP detection into routine practice.

First, extracellular vesicle isolation and characterization remain technically heterogeneous. Differences in sample collection, anticoagulant selection, centrifugation protocols, purification methods, and detection platforms may significantly influence measured vesicle concentrations and molecular profiles (11, 18).

Second, the lack of universally accepted BDMP-specific markers represents a major limitation. Although several neuronal, glial, and endothelial markers have been proposed to identify vesicle origin, no standardized combination of markers has been validated for routine clinical application (11, 78).

Third, current BDMP detection approaches, including advanced flow cytometry, nanoparticle-based analysis, and molecular characterization techniques, generally require specialized equipment and trained personnel. These technical requirements limit rapid bedside implementation, particularly in emergency settings where timely decision-making is critical (11, 25).

Therefore, before BDMP-based assays can be incorporated into acute TBI management, standardized detection protocols, quality-control procedures, and clinically validated reference ranges are required (11, 78).

### 4.5 Integration of BDMP biomarkers with conventional coagulation assessment

Given the complexity of TBI-IC, BDMPs are unlikely to replace existing coagulation assessments. Instead, they may serve as complementary biomarkers that provide additional information regarding cellular injury and biological mechanisms.

Current clinical evaluation of TBI-associated coagulation abnormalities relies mainly on conventional laboratory tests, including prothrombin time (PT), activated partial thromboplastin time (APTT), fibrinogen concentration, platelet count, and viscoelastic hemostatic assays (9, 79). These methods reflect systemic coagulation status but provide limited information regarding upstream cellular events (79).

BDMP measurement may theoretically complement these approaches by linking cellular injury signals with coagulation phenotypes. For example, elevated BDMP levels combined with evidence of hypercoagulability or fibrinolytic abnormalities may identify patients with specific biological profiles who require closer monitoring.

However, such integrated diagnostic strategies remain exploratory. Future studies should determine whether BDMP-based algorithms provide incremental clinical benefit compared with existing risk assessment approaches (80, 82).

### 4.6 Proposed clinical algorithm for BDMP integration

Based on current evidence, a potential future framework for BDMP application in TBI management may involve integration with existing clinical evaluation rather than replacement of conventional assessment. Such an approach may combine BDMP biomarker profiles with clinical assessment, neuroimaging findings, and conventional coagulation parameters to improve biological characterization and risk stratification of TBI-IC (Figure 2). However, this proposed approach remains conceptual and requires validation through prospective clinical studies before clinical implementation.

**Figure 2:**
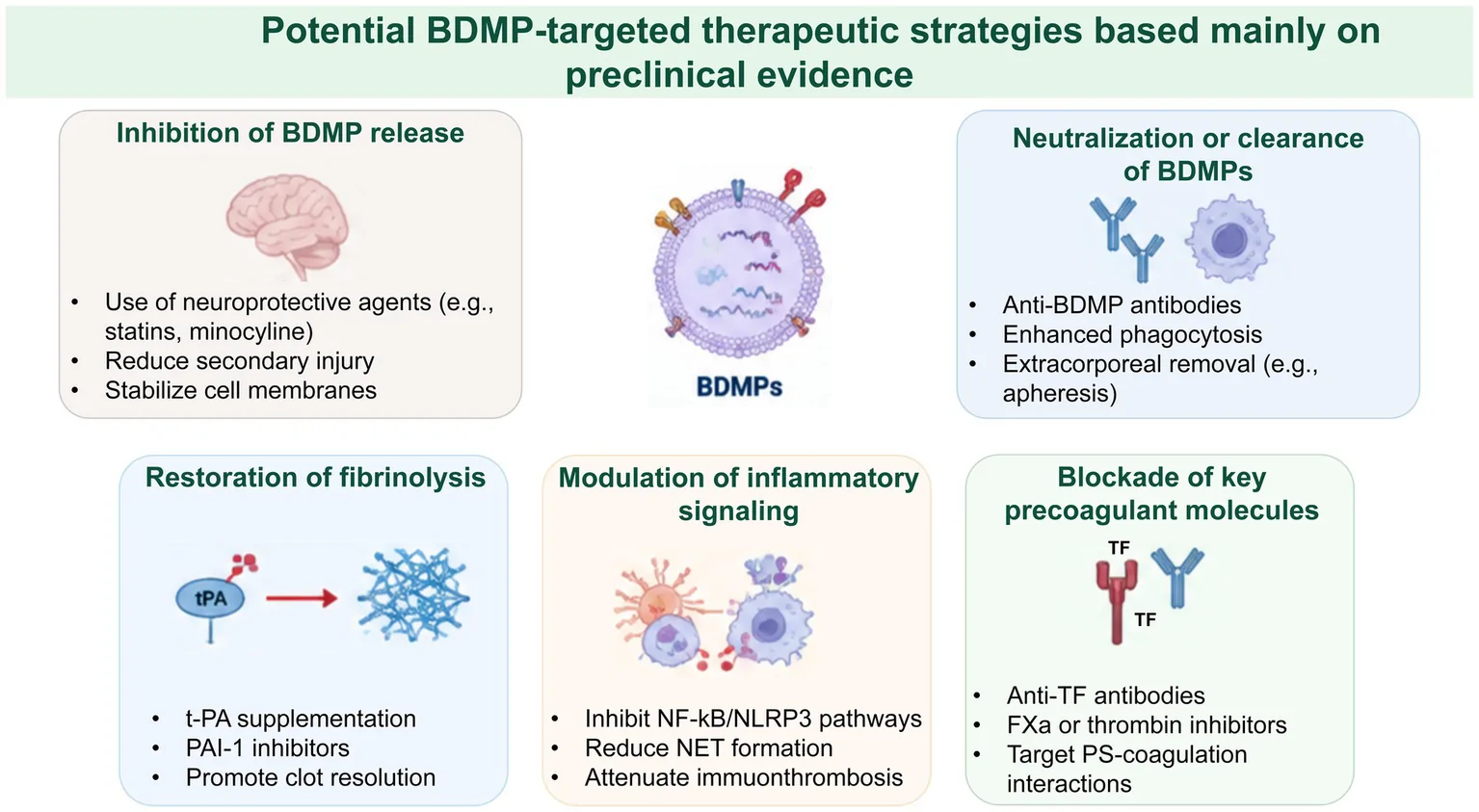
Potential BDMP-targeted therapeutic strategies based mainly on preclinical evidence. Potential therapeutic approaches include reducing BDMP generation, enhancing BDMP clearance, modulating BDMP-associated signaling pathways, and targeting specific molecular cargo. These strategies remain experimental and require further validation before clinical application.

Following admission of patients with suspected TBI, initial evaluation should continue to rely on neurological assessment, imaging findings, and routine coagulation testing. BDMP measurement, once standardized and clinically available, may provide additional biological information regarding cellular injury and coagulation risk (75, 79).

Patients with elevated BDMP levels combined with abnormal coagulation parameters may represent a subgroup with increased risk of secondary complications, potentially requiring intensified monitoring and individualized management (14, 51).

However, this proposed approach remains conceptual and requires validation through prospective clinical studies before clinical implementation.

## 5 Therapeutic implications

### 5.1 Current clinical management of TBI-IC

Management of TBI-IC remains focused on correcting abnormal hemostasis, preventing secondary bleeding, and maintaining adequate tissue perfusion (2). Current therapeutic strategies include blood component replacement, antifibrinolytic treatment, coagulation factor supplementation, and goal-directed coagulation management based on conventional laboratory tests or viscoelastic hemostatic assays (2, 8).

Blood product administration, including platelet transfusion, plasma supplementation, and fibrinogen replacement, is commonly considered in patients with clinically significant coagulation abnormalities or ongoing bleeding (83). Antifibrinolytic agents such as tranexamic acid (TXA) have demonstrated clinical benefits in selected trauma populations by reducing excessive fibrinolysis (84, 85). In addition, coagulation factor concentrates and viscoelastic-guided treatment strategies have been investigated to facilitate individualized management of trauma-induced coagulation abnormalities (80, 83).

However, current therapeutic approaches mainly target downstream coagulation consequences rather than upstream biological events associated with cellular injury. These interventions may correct abnormal coagulation parameters but do not specifically address the release of injury-associated extracellular vesicles or their potential effects on coagulation, inflammation, and vascular dysfunction.

The emerging understanding of BDMP biology provides a potential opportunity to explore upstream regulatory strategies. Nevertheless, BDMP-targeted interventions remain largely experimental, and their clinical applicability requires further investigation.

### 5.2 Emerging therapeutic strategies targeting BDMPs

BDMP-targeted approaches represent a potential therapeutic direction based on the hypothesis that modulating vesicle generation, clearance, signaling activity, or molecular cargo may reduce downstream pathological effects after TBI.

However, it should be emphasized that current evidence supporting BDMP-targeted therapy is primarily derived from experimental models. Whether these approaches can be safely and effectively translated into clinical practice remains unknown.

#### 5.2.1 Inhibition of BDMP generation

One potential strategy is to reduce excessive BDMP production following brain injury. Cellular stress responses, including calcium influx, cytoskeletal remodeling, and membrane instability, contribute to microparticle formation after injury (23, 86).

Experimental studies have investigated pharmacological inhibition of vesicle generation pathways as a means of reducing BDMP release (87). For example, MDL-28170, a calpain inhibitor, has been reported to decrease BDMP production and improve pathological outcomes in experimental models of TBI (14).

The potential mechanism may involve inhibition of calcium-dependent cytoskeletal disruption and reduced membrane shedding (22). However, because calpain signaling participates in multiple cellular processes, the effects of such interventions may not be specific to BDMP regulation. Furthermore, evidence supporting clinical efficacy in human TBI is currently unavailable.

Therefore, inhibition of BDMP generation remains a promising but exploratory strategy requiring further validation.

#### 5.2.2 Promotion of BDMP clearance

Another potential approach involves enhancing the removal of circulating BDMPs after their release into the bloodstream.

PS-exposing extracellular vesicles may be recognized and cleared by endogenous clearance mechanisms involving scavenger receptors and PS-binding proteins (88–92). Lactadherin, a PS-binding protein, has been investigated as a potential regulator of extracellular vesicle clearance (58).

Experimental studies suggest that lactadherin-mediated enhancement of PS-positive microparticle clearance may reduce coagulation abnormalities in animal models (58). However, the safety, specificity, and therapeutic feasibility of manipulating extracellular vesicle clearance pathways in patients with acute TBI remain unclear.

Because extracellular vesicles may also participate in physiological repair and immune regulation, excessive removal of BDMPs or other EV populations may have unintended biological consequences (11, 25).

#### 5.2.3 Blocking BDMP-mediated signaling pathways

Rather than eliminating BDMPs, another potential strategy is to interfere with pathological signaling pathways activated by BDMP interactions with recipient cells.

BDMPs may influence endothelial cells, immune cells, and other target cells through surface receptors and molecular cargo transfer (11, 20, 25). Among these pathways, sphingosine-1-phosphate receptor 2 (S1PR2)-related signaling has been implicated in endothelial dysfunction and blood-brain barrier disruption after traumatic brain injury, although whether BDMPs directly regulate this pathway remains unclear (69).

Experimental studies using pathway inhibitors such as JTE-013 have suggested that modulation of S1PR2 signaling may reduce endothelial injury and BBB disruption in experimental settings (69).

However, these findings should be interpreted cautiously. The effects of receptor inhibition may involve broader signaling networks rather than BDMP-specific pathways. Further studies are required to determine whether targeting BDMP-associated signaling provides therapeutic advantages over conventional anti-inflammatory or vascular-protective approaches.

#### 5.2.4 Targeting BDMP molecular cargo

Because BDMPs transport diverse biological molecules, targeting specific pathogenic cargo represents another theoretical therapeutic strategy.

Mitochondrial components, inflammatory mediators, coagulation-related proteins, and nucleic acids carried by BDMPs may contribute to downstream biological effects after TBI (46, 48, 49). Therefore, selectively blocking harmful cargo loading, release, or activity may provide a more precise therapeutic approach while preserving potentially beneficial extracellular vesicle functions.

However, current studies investigating cargo-targeted strategies remain at an early experimental stage. The identification of truly pathogenic BDMP components, development of selective targeting methods, and assessment of off-target effects represent major challenges before clinical translation.

### 5.3 Challenges in BDMP-targeted therapy

Despite increasing interest in BDMPs as therapeutic targets, several issues must be addressed before clinical application.

First, BDMP populations are heterogeneous, and not all BDMPs are necessarily harmful. Some extracellular vesicles may participate in tissue repair, immune regulation, and cellular communication (11, 17). Therefore, non-selective inhibition or elimination of BDMPs may interfere with physiological responses.

Second, the optimal therapeutic window remains unclear. Because BDMP release occurs dynamically after TBI, intervention timing may critically influence therapeutic outcomes. Strategies applied during early injury stages may differ from those appropriate during later phases characterized by immune regulation or fibrinolytic shutdown (5, 73).

Third, most current evidence is based on animal models. Differences between experimental TBI models and human injury patterns may limit direct clinical translation (93). Prospective clinical studies are required to determine whether BDMP modulation improves neurological outcomes without increasing adverse effects.

Overall, BDMP-targeted therapies represent an emerging research direction rather than established clinical interventions. Future studies should focus on identifying specific pathogenic BDMP subsets, developing standardized detection methods, and evaluating therapeutic efficacy in clinically relevant models. Potential BDMP-targeted therapeutic strategies are summarized in Figure 3.

**Figure 3:**
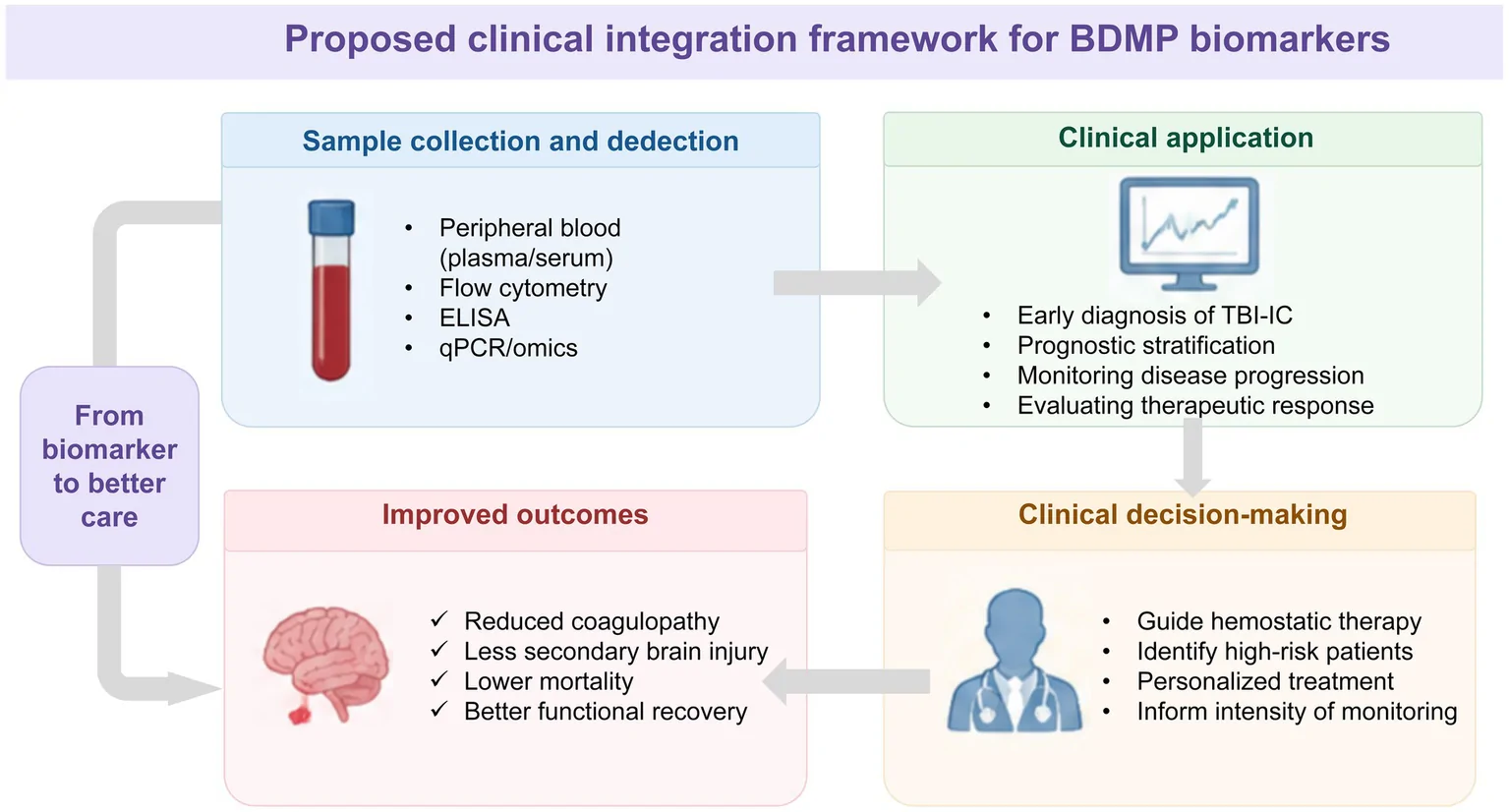
Proposed clinical integration framework for BDMP biomarkers. A proposed framework for integrating BDMP biomarkers into the clinical assessment of TBI-IC. Following standardized blood sampling and BDMP detection, biomarker information may support early risk assessment, prognostic stratification, disease monitoring, and evaluation of treatment response, thereby informing individualized clinical decision-making. Clinical utility remains to be established in prospective studies.

## 6 Challenges, limitations and future directions

Although increasing evidence suggests that BDMPs may participate in TBI-IC, several important challenges remain before their biological significance and clinical applicability can be fully established (14). Current understanding of BDMP function is limited by methodological heterogeneity, insufficient mechanistic validation, and the predominance of experimental evidence (11, 25). Addressing these limitations will be essential for determining whether BDMPs represent active mediators, biomarkers of injury, or both.

### 6.1 Lack of standardization in BDMP identification and quantification

One of the major challenges in BDMP research is the absence of standardized approaches for isolation, identification, and quantification.

Extracellular vesicle studies are influenced by multiple methodological factors, including sample collection, anticoagulant selection, isolation procedures, storage conditions, and detection platforms (11, 94). Differences in these experimental procedures may contribute to variability between studies and limit direct comparison of reported findings (11, 18).

Furthermore, the identification of BDMPs requires reliable markers capable of distinguishing brain-derived vesicles from other circulating extracellular vesicle populations (40). Although several neuronal, glial, and endothelial markers have been proposed, no universally accepted marker combination currently exists (11, 78).

The lack of standardized BDMP characterization criteria also complicates clinical translation. Before BDMP-based assays can be incorporated into clinical practice, consensus regarding detection methods, quality-control procedures, reference ranges, and reporting standards will be required (11, 17).

### 6.2 Temporal dynamics of BDMP release and functional changes

The temporal relationship between BDMP release and coagulation abnormalities after TBI remains incompletely understood.

Current experimental studies suggest that BDMP levels increase rapidly after injury, with early elevation occurring within several hours after TBI (13, 14). However, coagulation abnormalities may evolve over a longer period, including transitions between hyperfibrinolysis, coagulation factor consumption, hypercoagulability, and fibrinolytic shutdown (2, 6).

This temporal discrepancy raises an important question: do BDMPs directly initiate coagulation abnormalities, or do they represent an early indicator of cellular injury that subsequently influences downstream responses?

In addition, BDMP composition may change over time. Early-stage BDMPs released from damaged cells may contain procoagulant or inflammatory components, whereas later-stage vesicles may participate in immune regulation or tissue repair (11). Longitudinal studies are therefore needed to characterize BDMP dynamics throughout different phases of TBI.

### 6.3 Causality versus association

A major unresolved issue is whether BDMPs directly contribute to TBI-IC or primarily reflect the extent of tissue injury.

Clinical studies have demonstrated associations between circulating extracellular vesicle levels and injury severity; however, most studies have not specifically isolated BDMP populations or established causal relationships between BDMP elevation and coagulation abnormalities (13, 74).

Experimental studies provide mechanistic evidence that BDMPs may influence coagulation activation, platelet responses, inflammation, and endothelial dysfunction (14, 51). Nevertheless, many findings are derived from animal models or *in vitro* systems, where experimental conditions may not fully reproduce the complexity of human TBI.

Future studies should combine cell-specific BDMP characterization, functional experiments, and longitudinal clinical observations to determine whether BDMPs act as pathogenic mediators, disease indicators, or context-dependent regulators.

### 6.4 Translational challenges from experimental models to human TBI

Most current knowledge regarding BDMP-mediated mechanisms originates from animal models or cellular experiments. These studies have provided important insights into potential biological pathways but require cautious interpretation when applied to human disease (12, 14).

Experimental TBI models differ from clinical injury in several aspects, including injury mechanism, severity, systemic inflammatory response, and therapeutic intervention (95). In addition, differences in immune systems and coagulation regulation between species may influence BDMP generation and function.

Human studies investigating BDMPs remain relatively limited, and available evidence mainly focuses on circulating extracellular vesicle abundance or associations with clinical parameters (13, 75). Large-scale prospective studies are needed to determine whether BDMP-related measurements provide additional predictive value beyond established clinical indicators.

### 6.5 Clinical translation and future therapeutic development

The potential clinical application of BDMPs involves two major directions: biomarker development and therapeutic targeting.

As biomarkers, BDMPs may provide information regarding cellular injury and biological processes that are not captured by conventional coagulation tests. However, their clinical usefulness requires demonstration of improved diagnostic accuracy, prognostic performance, and cost-effectiveness compared with existing assessment strategies (75, 79).

As therapeutic targets, BDMPs represent an attractive but challenging concept. Strategies aimed at reducing BDMP generation, enhancing clearance, blocking pathological signaling, or modifying specific cargo remain largely experimental (14, 58). Future therapeutic development should consider several factors, including target specificity, optimal intervention timing, preservation of beneficial extracellular vesicle functions, and potential systemic effects (11, 25).

Importantly, BDMP-targeted interventions should not be viewed as replacements for established TBI management strategies. Instead, they may eventually serve as complementary approaches aimed at modifying upstream biological processes involved in secondary injury.

### 6.6 Future research priorities

Future investigations should focus on several key areas.

First, standardized methods for BDMP isolation and characterization should be established to improve reproducibility across studies (11, 17).

Second, advanced single-vesicle analysis and multi-omics approaches may help identify BDMP subpopulations with specific biological functions and distinguish pathogenic vesicles from potentially protective populations (17, 18, 96).

Third, longitudinal clinical studies are required to clarify the relationship between BDMP dynamics, coagulation phenotypes, neurological outcomes, and therapeutic responses (5, 13).

Fourth, mechanistic studies using clinically relevant models and human-derived samples are needed to determine whether BDMPs represent causal drivers of coagulopathy or secondary indicators of brain injury (14, 51).

Finally, integration of BDMP measurements with existing clinical parameters may provide a more comprehensive framework for individualized assessment and management of TBI-IC.

## 7 Conclusion

BDMPs represent an emerging component of the complex biological network linking brain injury and systemic coagulation abnormalities after TBI. Current evidence suggests that BDMPs may participate in coagulation regulation, platelet modulation, inflammatory responses, BBB dysfunction, and fibrinolytic imbalance through their membrane components and molecular cargo.

However, the current understanding of BDMP function remains incomplete. Most mechanistic evidence originates from experimental models, whereas human studies have primarily demonstrated associations rather than direct causality. Challenges including EV heterogeneity, lack of standardized detection methods, limited clinical validation, and uncertainty regarding therapeutic targeting currently restrict clinical translation.

Future studies integrating standardized BDMP characterization, longitudinal clinical investigation, and mechanistic validation are required to determine the precise role of BDMPs in TBI-IC. Rather than representing an established therapeutic target, BDMPs should currently be considered a promising research direction. Further studies are required to determine whether BDMP-based approaches can provide clinically meaningful benefits in TBI management.
